# A phase I clinical trial of adoptive T cell therapy using IL-12 secreting MUC-16^ecto^ directed chimeric antigen receptors for recurrent ovarian cancer

**DOI:** 10.1186/s12967-015-0460-x

**Published:** 2015-03-28

**Authors:** Mythili Koneru, Roisin O’Cearbhaill, Swati Pendharkar, David R Spriggs, Renier J Brentjens

**Affiliations:** Department of Medicine, Memorial Sloan Kettering Cancer Center, 1275 York Avenue, New York, NY 10065 USA; Weill Cornell Medical College, New York, NY USA

**Keywords:** Chimeric antigen receptors, IL-12, Ovarian cancer, Tumor microenvironment, MUC16

## Abstract

**Purpose:**

Recurrent platinum-resistant ovarian cancer has no curative options, necessitating the development of novel treatments, including immunotherapy.

**Rationale:**

Patient-derived T cells can be genetically modified to express chimeric antigen receptors (CARs) specific to tumor-associated antigens in an HLA-independent manner, with promising preclinical results. MUC16^ecto^ is highly expressed on most epithelial ovarian carcinomas but at low levels on normal tissues, offering an excellent immunotherapeutic target for this cancer. CAR T cells further modified to secrete IL-12 show enhanced cytotoxicity, persistence, and modulation of the tumor microenvironment.

**Design:**

We propose a dose escalation phase I clinical trial for patients with recurrent MUC-16^ecto+^ ovarian cancer to test the safety of intravenous and intraperitoneal administration and the preliminary efficacy of autologous IL-12 secreting, MUC-16^ecto^ CAR T cells containing a safety elimination gene.

**Innovation:**

This trial targets MUC-16^ecto^, a novel and promising tumor-associated antigen. This will be the first time CAR T cells are injected intraperitoneally directly into the site of the tumor within the abdomen in humans. Furthermore, the ability of genetically modified cells to secrete IL-12 will potentially enhance CAR T cell persistence and modulate the tumor microenvironment. For safety purposes, an elimination gene has been incorporated into the CAR T cells to mitigate any on-target, off-tumor or other unforeseen toxicity.

## Background and rationale

### CAR T cell therapy in ovarian cancer

Ovarian cancer is the second most common gynecologic malignancy in the United States, with 21,290 projected new cases in 2015, and the most lethal gynecologic malignancy, with 14,180 estimated deaths in the same year [1]. Due to the insidious nature of this disease, the majority of patients with ovarian cancer are diagnosed with advanced disease due to intraperitoneal spread and often the presence of distant metastases (International Federation of Gynecology and Obstetrics [FIGO] stage III/IV disease). The standard of care for these patients includes cytoreductive surgery, when feasible, followed by combination platinum- and taxane-based chemotherapy [2-4]. Despite the fact that up to 75% of patients achieve a good clinical response following initial therapy, almost all will ultimately relapse and eventually develop chemotherapy-refractory disease [5]. For this reason, novel therapeutic approaches are urgently needed.

Epithelial ovarian cancer appears well suited for the investigation of immunotherapeutic strategies, such as adoptive T cell therapy, given that the presence of tumor infiltrating lymphocytes (TILs) correlates positively with patient survival [6,7]. Chimeric antigen receptors (CARs) are artificial cell receptors that allow T cells to target a tumor-associated antigen (TAA). We and others have developed CAR genes in order to generate genetically modified tumor-specific T cells [8]. These CARs combine the targeted antigen specificity provided by monoclonal antibodies (mAbs) with the cytotoxic anti-tumor effects of T cells. Furthermore, these CARs are capable of recognizing TAAs in a human leukocyte antigen (HLA)-independent manner. Therefore, the same receptor may be applied to all patients, independent of HLA type or level of HLA expression by the tumor cell. Since the modified T cells are autologous to the patient, the risk of unforeseen graft-versus-host disease (GVHD) is largely averted.

Overcoming a hostile tumor environment is one of the major challenges facing adoptive T cell therapy. Various factors that cause dysfunction in TILs, such as regulatory T cells (Tregs) and inhibitory cytokines, can also negatively regulate *in vivo* persistence and antitumor activity of CAR T cells [9]. Though the infusion of CAR T cells can increase the proportion of functional T cells relative to suppressive Tregs, the rise in number alone may not be sufficient to overcome the inhibition. To this end, CAR T cells can be modified to secrete stimulatory factors that promote a productive anti-tumor immune response, even in the presence of suppressive Tregs and other inhibitory elements.

### Target, addition of IL-12 gene, elimination gene

**Figure 1:**
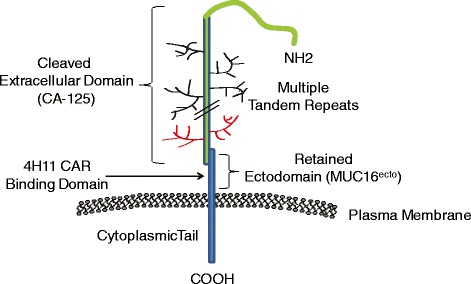
**Schematic diagram of MUC-16 structure.**

To create an effective CAR T cell, an appropriate target needs to be identified. The ovarian cancer antigen, MUC16, is over-expressed by a majority of ovarian cancers [10]. The recent isolation of the gene encoding MUC16 by the laboratory of Kenneth Lloyd [11] allowed for the characterization of MUC16 as a glycosylated mucin. Significantly, the full-length glycoprotein consists of a large cleaved and released domain termed CA-125 consisting of multiple repeat sequences, each containing a disulfide loop of 19 amino acids, followed by a retained cytoplasmic domain, MUC16^ecto^, which includes a residual non-repeating extracellular fragment, a transmembrane domain, and a cytoplasmic tail containing a phosphorylation site (Figure 1). CA-125, an FDA-approved tumor marker for ovarian cancer, is elevated in approximately 70-80% of women with epithelial ovarian cancer. To date, all reported mAbs to MUC16 bind to epitopes present on the released fraction of the glycoprotein, with none known to bind to the retained extracellular fraction. Since the MUC16^ecto^ fraction is retained on the cell surface and expressed only at low levels on normal tissue, including the uterus, fallopian tubes, ovaries and corneal surface of the eye, it is a highly attractive target for CAR-based adoptive T cell therapy [12-14]. A hybridoma that generates a mAb specific to the extracellular retained fraction of the MUC16 antigen (MUC16^ecto^) has been utilized to create a CAR specific to MUC16^ecto^ (4H11-28z), which in turn can be utilized to engineer autologous T cells targeted to the retained, surface-exposed antigen.

**Figure 2:**
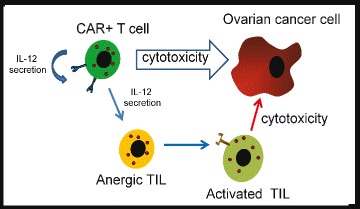
**Secretion of IL-12 by CAR-modified T cells may improve cytotoxicity of CAR** ^**+**^ **T cells and reverse anergy in tumor infiltrating lymphocytes.**

Though an appropriate target antigen is necessary, it may not be sufficient in creating an effective CAR against a solid tumor given the inhibitory tumor microenvironment. Therefore, we have armored the CAR with the ability to secrete interleukin-12 (IL-12), which can modulate the negative effects of the tumor microenvironment. IL-12 is a heterodimeric inflammatory cytokine expressed by activated antigen-presenting cells (APCs), neutrophils, and macrophages [15]. IL-12 is a potent inducer of a Th1 CD4^+^ T cell response and serves as a “signal 3” in concert with T cell receptor (TCR) activation (signal 1) and CD28 co-stimulation (signal 2) to CD8^+^ T cells, resulting in optimized clonal expansion and effector function [16]. IL-12 further induces proliferation and cytotoxic activity of natural killer (NK) cells and generates anti-tumor activity through effector cell production of cytokines, including interferon-gamma (INF-γ), which in turn up-regulates Fas (CD95) and FasL on tumor cells. More significantly, IL-12 has been shown to modulate the hostile tumor microenvironment through multiple mechanisms, including reactivation of anergic TILs, inhibition of Treg-mediated suppression of effector T cells, recruitment of NK cells to the tumor site, and inhibition of IL-10 and transforming growth factor beta (TGF-β) secretion by tumor-associated macrophages (TAMs) [17-19]. We have previously demonstrated in preclinical models that CAR-targeted T cells traffic to systemic sites of tumor involvement [20]. To this end, we predict that infusion of CAR T cells further modified to secrete IL-12 will result in targeted secretion of this cytokine within the tumor microenvironment. As a result, we further predict enhanced *in vivo* persistence and anti-tumor activity of these T cells, now resistant to inhibition by Tregs, with subsequent reactivation of anergic endogenous tumor-targeted T cells as well as IL-12 induced recruitment and activation of the innate tumor-targeted NK cells (Figure 2).

If on-target, off-tumor or unforeseen toxicities are encountered, the addition of a “safety switch” permits the removal of aberrant genetically modified T cells *in vivo* [21]. In preclinical models, the administration of cetuximab, an anti-EGFR (epidermal growth factor receptor) mAb that binds to the truncated EGFR (EGFRt) protein expressed by the CAR-modified T cells, induced death of EGFRt-transduced T cells via multiple mechanisms, including antibody-dependent cellular cytotoxicity and complement-mediated cytotoxicity [21]. In a published study by Wang *et al.,* greater than 50% of EGFRt^+^ T cells were killed *in vitro* within 1 hour after exposure to cetuximab; *in vivo,* NOD/*scid* mice engrafted with EGFRt^+^ T cells were cleared of those cells after 4–6 days of daily administration of cetuximab 1 mg intraperitoneally [21]. The use of cetuximab to eliminate EGFRt^+^ CAR-modified T cells is approved under BB-IND-15829.

Preclinical data using tricistronic vector indicates that 4H11-28z/fIL-12/EFGRt CAR T cells show enhanced proliferation and increased IFN-γ secretion compared to control CAR T cells *in vitro* [22]. In mouse models using human ovarian cancer xenografts, IL-12 secreting CAR T cells exhibit enhanced anti-tumor efficacy exemplified by increased survival, prolonged persistence of T cells, and higher systemic IL-12 and IFN-γ levels [22]. The addition of cetuximab causes >60% CAR T cell death after only 4 hours *in vitro,* while *in vivo*, the addition of cetuximab to mice injected with 4H11-28z/fIL-12/EFGRt CAR T cells shows decreased levels of IL-12 and IFN-γ nearly to baseline [22]. These promising preclinical results validate the function of the three components of the CAR.

### Trial design

This is a phase I clinical trial testing the safety of intravenous (IV) and intraperitoneal (IP) infusion (with or without prior cyclophosphamide chemotherapy) of genetically modified autologous T cells in patients with recurrent MUC16^ecto+^ ovarian, fallopian tube, or primary peritoneal cancer. These autologous T cells will be genetically engineered to express the 4H11-28z CAR targeting the MUC16^ecto^ antigen, secrete IL-12, and express EGFRt elimination vector (4H11-28z/fIL-12/EFGRt). Patients with recurrent high-grade serous ovarian, primary peritoneal or fallopian tube carcinoma shown to express MUC16^ecto^ antigen confirmed by immunohistochemistry (IHC) analysis of banked (paraffin embedded) or freshly biopsied tumor will potentially be eligible for the study. A scoring system of 0–5 will be applied to MUC16^ecto^ expression as described by Dharma *et al.* that takes into account percentage of cells, intensity, and pattern of staining [23]. Only moderate to strong immunoreactive scores (3–5) will be considered positive, with a score of 3 described as 51-75% strong or 51-100% weak, 4 as 76-99% strong, and 5 as 100% strong staining. All patients will have received prior chemotherapy for recurrence, with a maximum of five prior lines of chemotherapy permitted. Patients with other active malignancies, a life expectancy of <3 months, or a Karnofsky Performance Status (KPS) score <70% at the time of planned treatment will be ineligible.

This is a standard phase I dose-escalation trial. Cohorts of 3–6 patients will be infused with escalating doses of modified T cells to establish the maximum tolerated dose (MTD). There are four planned dose levels: 3 × 10^5^, 1 × 10^6^, 3 × 10^6^, and 1 × 10^7^ 4H11-28z/fIL-12/EFGRt T cells/kg. Cohorts I and II will be treated with 3 × 10^5^ 4H11-28z/fIL-12/EFGRt T cells/kg but patients in cohort II will also receive lymphodepleting cyclophosphamide. Cohorts II-V will receive escalating doses of the modified T cells following pretreatment with cyclophosphamide. Lymphodepleting cyclophosphamide dosed at 750 mg/m^2^ will be administered 2–4 days prior to the initial T cell infusion. A standard 3 + 3 dose escalation schema will be followed. If the first dose level exceeds the MTD, a subsequent cohort of 3–6 patients will be treated at the −1 dose level of 1 × 10^5^ 4H11-28z/fIL-12/EFGRt T cells/kg without the addition of lymphodepleting cyclophosphamide (cohort -I) (Table 1).

**Table 1:** **4H11-28z/fIL-12/EFGRt T cell dose escalation**

**Cohort**	**Dose level**	**Cyclophosphamide dose**	**4H11-28z/fIL-12/EFGRt T cell dose**	**Number of patients**
-I	−1	None	1 × 10^5^ cells/kg	3-6 patients
I	1	None	3 × 10^5^ cells/kg	3-6 patients
II	1	750 mg/m^2^	3 × 10^5^ cells/kg	3-6 patients
III	2	750 mg/m^2^	1 × 10^6^ cells/kg	3-6 patients
IV	3	750 mg/m^2^	3 × 10^6^ cells/kg	3-6 patients
V	4	750 mg/m^2^	1 × 10^7^ cells/kg	3-6 patients

#### Intraperitoneal administration of CAR T cells

Studies in ovarian cancer have shown that chemotherapy regimens that incorporate IP delivery of treatment are superior to those of IV administration alone, which is rational given that the predominant site of ovarian cancer is within the peritoneal cavity. More specifically, several multicenter randomized clinical trials demonstrated increased progression-free and overall survival for IV/IP versus IV administration of chemotherapy. IV/IP chemotherapy is now considered standard of care in the first-line treatment of optimally debulked stage III ovarian cancer [24]. It is therefore reasonable to hypothesize that IP delivery of modified CAR T cell therapy may also lead to improved efficacy.

In this trial, we plan to deliver CAR T cell therapy via IV and IP routes. There is no previous human experience with IP delivery of CAR T cells, since the majority of CAR T cell therapies to date has been in patients with hematological malignancies; hence, we must rely on preclinical data from our mouse models. In Chekmasova *et al.* [25], SCID-Beige mice injected IP with human OV-CAR3(MUC-CD) tumor cells and then treated either IV or IP with 4H11-28z T cells showed statistically enhanced survival compared to control mice (who were either untreated or treated with CAR T cells targeting the irrelevant antigen CD19). However, IV- and IP-treated mice exhibited statistically equivalent antitumor efficacy using the second generation CAR (4H11-28z). We have further compared IV versus IP administration using IL-12 secreting CAR T cells and have found that while both groups of mice have extended survival, IP administration has improved survival compared to the IV route [22]. In this experiment, both groups of mice were initially injected with ovarian tumor (SKOV3 expressing MUC16^ecto^, 1 × 10^7^, IP) followed by CAR T cell injection either IP or IV (14 days later, 2.5 × 10^6^ CAR T cells). In this mouse model, GFP-luciferase positive tumor is injected IP, and subsequent bioluminescence imaging confirms that the engrafted tumors are restricted to the peritoneal cavity. These preclinical studies indicate that IP injections of CAR T cells are more efficacious than IV alone. Although the administration of IP chemotherapy in ovarian cancer patients reduces the risk of recurrence within the abdomen, these patients frequently have recurrences outside of the abdominal cavity [26,27]*.* Therefore, in our study, in addition to IP administration of the modified T cells we have also included IV dosing in order to address the extraperitoneal sites of disease.

#### Objectives and endpoints of the phase I clinical trial

The primary objective of this study is to assess the safety of IV and IP infusion of autologous 4H11-28z/fIL-12/EFGRt T cells with or without prior high-dose cyclophosphamide. The secondary objectives are to assess the anti-tumor efficacy and the *in vivo* persistence of adoptively transferred 4H11-28z/fIL-12/EFGRt T cells and finally to assess whether infusion of modified T cells enhances expansion of endogenous tumor-targeted T cells.

#### Regimen

Patients with recurrent high-grade serous ovarian cancer will be offered a screening informed consent to test their tumor for the expression of MUC16^ecto^. If MUC16^ecto^ positivity is confirmed by IHC (score 3–5, as described above under Trial Design), then patients will have a leukapheresis product obtained from peripheral blood. The leukapheresis product will be washed to remove excess platelet and red blood cell contamination and then frozen. In the treatment phase of the study, the leukapheresis product will be thawed and washed. Subsequently, CD3^+^ T cells will be isolated from the thawed leukapheresis product by magnetic separation using CD3/CD28 beads in the Cell Therapy and Cell Engineering Facility at MSKCC. Activated T cells will be retrovirally transduced with the MUC16^ecto^ specific 4H11-28z chimeric receptor, IL-12 gene, and EGFRt elimination gene and further expanded using an established CD3/CD28 bead expansion protocol.

Peripheral T cells will likely be quantitated by flow cytometry analysis using CD3-specific antibody to verify that a sufficient cell number is obtained. Although we anticipate achieving the desired T cell dose through our proposed transduction/expansion protocol for most patient samples, in cases where at least 50% of the planned T cell dose is not achieved following T cell stimulation, the patient will not be treated on the study and will be replaced by the next enrolled subject for that cohort.

When the modified T cells are ready for administration, all patients will have an IP catheter placed prior to T cell infusion. Patients will be admitted as inpatients to our hospital prior to their first infusion of CAR T cells and will remain hospitalized until at least 2 days after the second infusion of CAR T cells. The first cohort of patients to be treated, and the first patient treated in each subsequent cohort, will be admitted to the intensive care unit (ICU); subsequent patients may be admitted to the gynecologic medical oncology inpatient service (subject to the clinical judgment of the treating physician).

**Figure 3:**
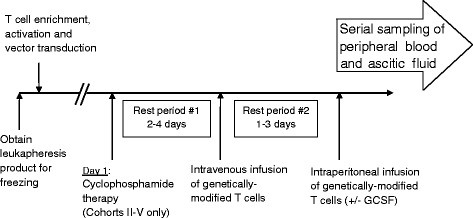
**Treatment schema.**

Patients treated in cohorts II-V will receive a single dose of lymphodepleting cyclophosphamide (750 mg/m^2^ IV) chemotherapy 2 to 4 days prior to initiating treatment with modified T cells. Prior to infusion, the transduced T cells will be quality tested for number, purity, viability, and sterility. The following T cell release criteria must be met, including viability >80%, CD3^+^ ≥95% with a minimum of 50% of planned T cell dose achieved according to CAR and EGFRt co-expression, and the infused T cell population must have a transduced fraction of >20% based on flow cytometric analysis of expanded T cell population. Furthermore, the average vector copy number in the transduced T cells will be determined by real time PCR before infusion and will need to be in the range of 0.25 to 4 copies per cell, and PCR will be used to ensure the absence of replication competent retrovirus in the transduced T cells. All patients will receive 50% of the genetically modified T cell dose intravenously. Patients will be closely monitored for toxicities. One to 3 days later, the remaining dose of T cells will be administered as an IP infusion (Figure 3). At least 3 patients will be treated at dose level 1, with an accrual of no more than 2 patients per month within each dose level. At least one week will elapse between treatments of each patient enrolled. All patients treated in the preceding cohort will be observed for a minimum of 4 weeks from the day of the initial T cell infusion before escalation to the next cohort occurs. In light of a significant risk for neutropenia (ANC ≤ 1,000/mm^3^) following cyclophosphamide therapy, patients treated with cyclophosphamide may be treated with growth factor support at the discretion of the investigators (either a single subcutaneous injection of pegfilgrastim or 3 consecutive days of subcutaneous filgrastim).

Blood samples will be obtained from all patients prior to and following treatment to assess toxicity, therapeutic effects, and survival of the genetically modified T cells. Post-treatment blood samples will be collected at approximately 1 hr, 1 day, and at 1, 2, 3, 4, 5, 6, 7, 8 and 12 weeks post T cell infusion, then monthly thereafter until 1 year, then yearly thereafter up to 15 years post T-cell infusions. If technically feasible, patients’ ascitic fluid may also be sampled prior to cyclophosphamide or T cell therapy (whichever comes first), as well as during follow-up. Patients will have CT scans at approximately 6 weeks, 3 months, 6 months, 9 months and 12 months after T cell infusion, and thereafter if clinically indicated.

#### Management of severe cytokine release syndrome (sCRS)

In ongoing trials of CAR-modified T cell therapy for patients with hematological malignancies, some patients with B cell acute lymphoblastic leukemia (B-ALL) who received modified T cells with a different construct (19-28z, which targets the CD19 antigen found on B cells) developed severe cytokine release syndrome (sCRS), which is characterized by persistent fever, elevation of serum cytokines, and clinical toxicities [28]. All cases of sCRS were observed in patients with morphologic B-ALL at the time of CAR-modified T cell infusion, and we anticipate the potential for similar toxicity for this ovarian cancer CAR T cell trial. Severe CRS is defined by the presence of one of the following clinical and laboratory parameters: (1) hypotension defined as systolic blood pressure <90 mmHg refractory to IVF challenge or requiring vasopressors, (2) respiratory distress/hypoxia requiring increasing supplemental oxygen or ventilatory support, (3) acute coronary syndrome with positive troponin and/or EKG changes, and (4) seizure clinically suspected and/or documented on EEG. Based on current ongoing trials with CAR T cells using the 19-28z construct, our experience is that sCRS manifests with fevers, hypotension, and CNS toxicity occurring at approximately day 4–5 post CAR T cell infusion [29].

We also anticipate potential toxicities related to IL-12, but these toxicities are more typically related to liver dysfunction and occur 1–3 days post-infusion [30,31]. Patients will be closely monitored, initially in the ICU, and also have regular blood work including assessments of hepatic and renal function. We will also monitor C-reactive protein (CRP) and cytokine levels (EGF, FGF-2, Eotaxin, TGF-a, G-CSF, Flt-3 L, GM-CSF, FRACTALKINE, IFN-a2, IFN-g, GRO, IL-10, MCP-3, IL-12p40, MDC, IL-12p70, IL-13, IL-15, sCD40L, IL-17, IL-1ra, IL-1a, IL-9, IL-1b, IL-2, IL-3, IL-4, IL-5, IL-6, IL-7, IL-8, IP-10, MCP-1, MIP-1a, MIP-1b, TNF-a, TNF-b, and VEGF). Our experience from the ongoing clinical trial using CARs to treat patients with leukemia has shown CRP levels to be useful in CRS [32]*.* Thirdly, we have a comprehensive algorithm that outlines the proposed treatment for patients who encounter CAR T cell toxicity including tocilizumab, cetuximab, and steroids.

**Figure 4:**
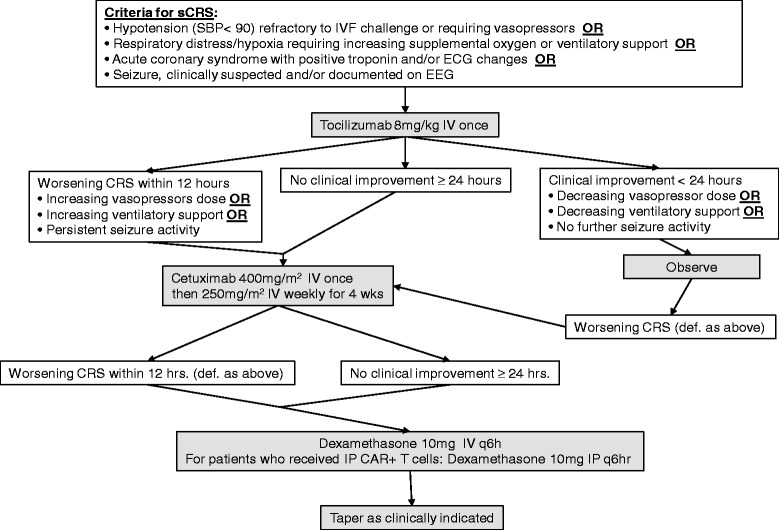
**Severe cytokine release syndrome management algorithm.**

Patients with sCRS will initially receive tocilizumab, a humanized mAb against IL-6 receptor. If the sCRS fails to resolve, then cetuximab will be administered IV to eliminate EGFRt CAR-modified T cells. Patients who show worsening CRS within 12 hours or who fail to improve clinically after 24 hours following administration of cetuximab will be treated with IV and IP dexamethasone if clinically indicated. Due to its potent and non-selective immunosuppressive activity, the administration of dexamethasone is expected to result in the elimination of any remaining CAR-modified T cells persisting in the patient following treatment with cetuximab (Figure 4) [33-35].

#### Statistical methods

There are four planned dose levels of 4H11-28z/fIL-12/EFGRt T cells: 3 × 10^5^, 1 × 10^6^, 3 × 10^6^, and 1 × 10^6^ 4H11-28z/fIL-12/EFGRt T cells/kg. The first subject will be treated at dose level 1 (3 × 10^5^ 4H11-28z/fIL-12/EFGRt T cells/kg). At least one week will elapse from the first patient’s T cell infusions before the second patient is treated (on dose level 1) to allow for toxicity and safetyassessment. Three subjects will be enrolled in cohort I and followed for 30 days for safety assessments. If no dose-limiting toxicity (DLT) is observed after all 3 subjects have been observed for 30 days, a second cohort of 3 subjects will be enrolled at the same dose level (3 × 10^5^ 4H11-28z/fIL-12/EFGRt T cells/kg) with the addition of cyclophosphamide. Cohorts will continue to be enrolled and observed at sequentially increasing (predefined) dose levels until 1 subject experiences a DLT. The MTD will then be defined using traditional dose escalation rules. If 2 of the first 2 subjects have a DLT, the enrollment of a third subject at the same dose level is not necessary. Similarly, if 2 of 4 subjects or 2 of 5 subjects have a DLT, it is not necessary to enroll subsequent subjects at the same dose level. This escalation/de-escalation will continue until the following conditions are met: either 6 subjects have been treated in the current cohort with no more than one DLT, or 2 or more subjects have a DLT at the higher cohort. When these conditions are met, the current dose level will be considered the phase II dose.

The secondary aims of the study will be addressed by descriptive exploratory statistical analyses, since the sample size is not known in advance and there are no formal hypotheses being tested. These analyses may include descriptions of time patterns for continuous variables measured repeatedly, both on the individual level and aggregated by dose level. Persistence of adoptively transferred 4H11-28z/fIL-12/EFGRt T cells will be assessed by RT-PCR analysis and by FACS at different time points, as described below, where we will obtain cell numbers and percentages of CAR T cells compared to endogenous T cells in pre- and post-treatment samples. Peripheral blood samples obtained prior to T cell infusions will be compared to peripheral blood samples 1-hour post T cell infusion and the samples taken daily for 2 days and weekly thereafter for 8 weeks. Samples will be analyzed using flow cytometry with a mAb specific to the 4H11-28z CAR and by quantitative RT-PCR analysis using primers and a probe targeting retroviral specific sequences. These peripheral blood samples will also be utilized to assess expansion of endogenous tumor-targeted T cells by infused 4H11-28z/fIL-12/EFGRt T cells by exclusion of modified T cells by FACS sorting. The remaining T cells will be analyzed for reactivity to tumor-specific targets by ELISPOT and multiparameter flow cytometric analyses. These experiments will provide a numerical count of endogenous tumor-specific T cells, which will be monitored and presented graphically.

Progression-free and overall survival will be described by the Kaplan-Meier method. Patients’ best overall radiographic response will be tabulated based on immune-related response criteria [36].

#### Translational studies

- Assess *in vivo* persistence of genetically modified T cells and characterize the endogenous tumor-specific T cell expansion.
  ***Study design:*** Following infusion, the presence of 4H11-28z/fIL-12/EFGRt autologous T cells in peripheral blood and ascites will be analyzed by flow cytometry using a fluorochrome-conjugated Armenian hamster mAb specific to the 4H11-28z CAR, and by Quantitative Real Time-PCR analysis using primers and probe targeting retroviral specific sequences. To assess whether infusion of IL-12 secreting tumor-targeted T cells enhance proliferation of endogenous tumor-specific T cells, peripheral blood samples will be obtained from patients on day 3 prior to infusion of modified T cells, and again 1 hour after infusion. Samples of peripheral blood and peritoneal lavage will also be drawn during follow-up. Briefly, these samples will be drawn daily for 2 days after T cell infusion, weekly thereafter for 8 weeks, and then monthly for 6 months. T cells will be isolated from these samples and sorted by FACS to exclude CAR T cells. The resulting T cell samples will be analyzed for reactivity to previously isolated tumor cells by ELISPOT and multiparameter flow cytometric analyses. In addition, we will assess phenotype of CAR T cells and endogenous T cells for effector versus memory cell markers in peripheral blood and ascites samples.
- Examine the tumor microenvironment following infusion of CAR T cells, including cytokine levels, T cell suppressing ligands (PD-1 and CTLA-4), and the presence of inhibitory cell types such as Tregs, TAMs, and myeloid-derived suppressor cells.
  ***Study design***: These samples at the previously discussed time points will be analyzed for levels of various cytokines, including IL-12 and IFN-γ, along with inhibitory cytokines such as IL-10 as measured by Luminex 100IS technology. The presence of inhibitory cell types such as Tregs, TAMs, and myeloid-derived suppressor cells will be assessed by flow cytometry using antibodies to appropriate surface antigens associated with these cell types. Lastly, flow cytometry will also be used to characterize the levels of T cell suppressing ligands such as PD-1 and CTLA-4 using antibodies for the pre- and post-infusion samples.

### Innovation

This trial offers several innovative features compared to previous trials utilizing CARs for ovarian cancer. To begin with, this trial incorporates a novel ovarian cancer antigen, MUC-16^ecto^. Secondly, the trial is the first to incorporate the addition of a cytokine to CARs allowing delivery of an immunostimulatory agent to the tumor microenvironment. In addition, the trial is the first to incorporate the use of tricistronic vector allowing for expression of three different genes, including EGFRt, IL-12, and the CAR itself (4H11-28z). Lastly, the route of CAR T cell administration, the combination of IP and IV injections, is unique to this trial.

The 4H11 antibody from which the CAR was derived has already been studied extensively against human high-grade serous ovarian carcinomas in comparison to normal tissues [23]. Tissue microarray slides representing primary, metastatic, and recurrent tumors demonstrated that the 4H11 antibody bound to high-grade serous ovarian tumors with high specificity. Normal tissues such as adult colon, rectum, ectocervix, small intestine, ovary, liver, pancreatic ducts, spleen, kidney and skin (as well as fetal heart, gallbladder, colon, small intestine, liver, rectum, adrenal, thyroid, spleen, skin, bone, epididymis, brain, lung, muscle, smooth muscle, kidney, eye, umbilical cord, and placenta) did not stain with this antibody [23]. The antibody for 4H11 did stain the luminal side of esophageal glands and a few other areas, such as bronchial epithelium, endocervical glands, and gastric glands, but only in the cytoplasm [23]. Although MUC16 expression has been found in the superficial epithelium at the corneal surface of the eye, the antibody from which our CAR is derived did not stain in the eye [14,23]. Therefore, due to the limited staining of the 4H11 antibody on the extracellular surface of normal cells and the high specificity to serous ovarian tumors, the 4H11 CAR is a promising target for use in a phase I clinical trial.

Given concern for the lack of *in vivo* persistence of CAR T cells and the potential impairment by hostile tumor microenvironment, we have further modified the CAR T cells to secrete IL-12, a stimulatory cytokine that can promote an anti-tumor response through multiple mechanisms, including enhanced proliferation and CD8+ T cell effector function. However, we acknowledge the potential toxicity of IL-12 secreting CAR T cells. We appreciate the importance of locoregional delivery of IL-12 as a mechanism to limit toxicity since the CAR will target the T cell to the site of tumor. In addition, our preclinical studies indicate that the amounts of IL-12 we observe both *in vitro* and *in vivo* mouse models are lower than those observed by other groups [22,37,38]. Studies using both syngeneic and xenotransplant mouse models have not demonstrated systemic toxicity, as reported by others, and this has been confirmed by pathology reports and formal toxicology studies [22]. Furthermore, in our *in vivo* model, we have found once the antigen has been eradicated, IL-12 levels decreased to nearly undetectable levels [22]. The IL-12 levels published in humans trials (>2000 pg/mL) are 40-fold higher than the values in our murine studies (<50 pg/mL), which is anticipated to reduce deleterious effects [22,31,39]. Looking at the production of IL-12 *in vitro*, the levels of IL-12 by CAR T cells are >5-fold lower compared to CAR expressing flexi-IL12 or NFAT IL-12 from a publication from the NIH when we compared the levels seen with this system compared to the amounts described in that publication from the NIH [37]*.* Though previous studies have indicated toxicity with IL-12, we believe that the lower levels associated with this construct will correlate with less toxicity but still provide necessary added benefit. The lower IL-12 levels observed are likely due to the fact that the IL-12 is located after an IRES element and that the construct is tricistronic. Given the concern that there may be differences in the sensitivity of mice versus humans on IL-12–mediated systemic toxicity, we have included several safety measures in our study design to mitigate the risks to patients.

Firstly, we will start at a much lower starting dose than used in the leukemia CAR T cell trials that are currently underway at our institution (NCT00466531, NCT01860937). The starting dose in our trial will be 3 × 10^5^ CAR T cells/kg compared to 3 × 10^6^ CAR T cells/kg in the leukemia trials. Secondly, we have incorporated an elimination gene in the vector. Thirdly, our experience of CAR technology in leukemia has enabled us to develop a comprehensive algorithm that outlines the proposed treatment for patients who encounter CAR T cell toxicity. If the patient fulfills the criteria for sCRS, then the patient will initially be dosed with tocilizumab. However, if the patient does not improve or develops worsening CRS, the EGFRt elimination gene can be activated through administration of IV cetuximab. In the event of further deterioration, the presence of the IP catheter means that patients will also be able to receive dexamethasone IP as well as IV.

We propose that split IV/IP route dosing is appropriate for 2 reasons: (1) IV dosing addresses metastatic disease outside of the peritoneum, and (2) IP administration has been shown in our murine models to have more long-term efficacy compared to IV administration. The 1 to 3 day interval between the IV and IP T cell infusions will permit us to monitor patients for any toxicity related to the CAR T cells before proceeding to the first in-human IP administration of CAR T cells. Additionally, we have performed toxicology studies in which half the dose was given IV and half IP in the same group of mice. In this study, complete necropsy and blood work has been completed. The results indicate no adverse events or toxic lesions in an immune competent syngeneic mouse model after CAR administration 2 weeks post-tumor injection at a dose of 2.5 million CAR T cells IV followed by next day delivery of 2.5 million CAR T cells IP (data not shown).

## Discussion

Adoptive immunotherapy with CAR autologous T cells is a new approach in the treatment of advanced ovarian cancer allowing for targeted antitumor activity with the hope of providing prolonged disease control through potential modulation of the endogenous immune system. The use of CARs has been successful in early clinical trials in patients with B-cell leukemias. Recently, our group published the successful results of a phase I adult ALL trial targeting CD19^+^ tumors using (19-28z) CAR T cells [29]. However, trials utilizing CAR therapy in ovarian cancer are limited and to date have not been successful [40].

Our goal is to determine the safety and feasibility of IV and IP infusion of autologous 4H11-28z/fIL-12/EGFRt T cells with or without prior high-dose cyclophosphamide. Ultimately, we hope this approach will lead to *in vivo* persistence of adoptively transferred 4H11-28z/fIL-12/EFGRt T cells allowing for anti-tumor efficacy and further modulation of tumor microenvironment, such that the patient develops increased progression-free survival. Choosing the appropriate target antigen is one of the initial steps in engineering an improved CAR to accomplish these objectives. MUC16^ecto^ is the retained extracellular portion of epithelial ovarian carcinoma, which is a TAA that the CAR targets. The antibody from which the CAR is derived has been extensively tested in human ovarian models and normal tissues, showing high specificity with low background levels [23]. We use CD28 as a co-stimulatory domain along with CD3ζ trans membrane protein in a gammaretroviral backbone, which acts as a potent amplifying signal for CD19 specific CARs in the clinical setting. Therefore, we incorporated a similar design in the ovarian CAR.

This clinical trial is unique in numerous other aspects compared to previous trials, mainly due to the incorporation of CAR T cells that secrete IL-12, allowing for the favorable modulation of the tumor microenvironment and the endogenous immune system. Other novel features include a different target antigen, MUC-16^ecto^, the tricistronic nature of the construct, and the inclusion of IP administration of CAR T cells. Though numerous CARs have been designed to target TAAs, including α-folate receptor, NKG2D, and mesothelin, only the results of the trial utilizing the α-folate receptor have been published, and they show a lack of CAR T cell persistence [40-43]. We hope to overcome this limitation by the addition of IL-12 to allow for enhanced persistence, which has been a noted feature in orthotopic human ovarian mouse models [22]. A recent publication from the NIH using TILs genetically engineered with an inducible IL-12 for metastatic melanoma shows the utility of IL-12 in solid tumors allowing for the absence of IL-2 administration and lower cell doses than trials using conventional TILs [39]. However, the authors discussed toxicities observed at higher doses of 0.3 to 3 × 10^9^ NFAT.IL-12 cells, including hepatic dysfunction, high fevers and periodic hemodynamic instability, which they attributed to the IL-12. In addition, they mentioned the lack of proliferation and persistence of IL-12 secreting TILs. Our pre-clinical studies indicate that IL-12 secreting CAR T cells show improved proliferation compared to non-IL-12 secreting CAR T cells *in vitro* and show enhanced persistence in murine tumor models [22]. Furthermore, as supported by our preclinical studies, we believe that our approach will be associated with less IL-12 related toxicity. We anticipate lower IL-12 levels for 3 reasons: 1) the IL-12 gene follows an IRES element, 2) the tricistronic nature of the construct, and 3) the lower CAR T cell doses used. As an added safety feature we have incorporated an elimination gene. Moreover, patients will be closely monitored in the inpatient setting during and following treatment with CAR T cells. Enrollment on the study will be staggered both within and between cohorts to allow for toxicity assessment.

Adoptive immunotherapy has shown promise in hematologic malignancies. Recently, we published the successful results of phase I adult ALL trial targeting CD19^+^ tumors using (19-28z) CAR T cells [29]. The surge for better treatment continues as CAR-T therapy enters the solid tumor arena. We propose a novel phase I ovarian cancer trial targeting MUC-16^ecto^ using 4H11 antibody. It is highly specific for epithelial ovarian carcinomas without being significantly expressed on other normal tissues. Carrying forward the tradition of armored CARs, we incorporated IL-12 transgene into our vector design to alter the tumor milieu and educate immune cells within the hostile tumor microenvironment to fight against the tumor along with effector T cells and CARs while making them persist longer. Safety has been a major issue of concern while using CAR T therapy over the years. We incorporated EGFRt gene into the construct, which will cause the cells to be eliminated upon administration of cetuximab, thereby creating a “safety switch”. Intraperitoneal administration of CAR T therapy along with conventionally used IV infusion presents a unique technique, as it ensures tumor killing within the peritoneum, along with any distant metastasis.

## Conclusions

In summary, we propose that this clinical trial using CAR technology for patients with advanced recurrent MUC16^ecto+^ ovarian, fallopian tube, or primary peritoneal cancer will be beneficial overall compared to the potential risks. Given the natural history of advanced-stage ovarian cancer, characterized by multiple recurrences and invariable development of chemotherapy-refractory disease, alternative therapies are required. Though numerous clinical trials have shown utility of CAR T cell therapy in hematological malignancies, it remains unclear whether such an approach would have the same efficacy in solid tumors. We developed this trial with particular attention to address potential limitations of the few other unsuccessful CAR trials in solid tumors. The main principle difference with this trial compared to others is the concept of using the CAR T cell not only as a mechanism to enhance tumor-directed cytolysis but also to act as a delivery tool of an agent that can further modulate the tumor microenvironment. Localized delivery of IL-12 to the tumor microenvironment can enhance the endogenous immune system and allow for further participation in tumor elimination. While engineering of CAR-expressing T cells is a relatively novel field, particularly with respect to solid malignancies, if successful, this approach could eventually be extended to a wide variety of other tumor types.

## Abbreviations

FIGO: International Federation of Gynecology and Obstetrics
CAR: Chimeric antigen receptor
TAA: Tumor-associated antigen
mAb: Monoclonal antibody
HLA: Human leukocyte antigen
GVHD: Graft-versus-host disease
TCR: T cell receptor
APCs: Antigen-presenting cells
NK: Natural killer
INF-γ: Interferon-gamma
TAMs: Tumor-associated macrophages
TGF-β: Transforming growth factor beta
Tregs: Regulatory T cells
scFv: Single-chain fragment antibody
EOC: Epithelial ovarian cancer
EGFR: Epidermal growth factor receptor
EGFRt: Truncated epidermal growth factor receptor
IL-12: Interleukin-12
IP: Intraperitoneal
IV: Intravenous
TILs: Tumor-infiltrating lymphocytes
IHC: Immunohistochemistry
KPS: Karnofsky Performance Status
MTD: Maximum tolerated dose
B-ALL: B cell acute lymphoblastic leukemia
sCRS: Severe Cytokine Release Syndrome
CRP: C-reactive protein
DLT: Dose-limiting toxicity

## References

[1] Siegel RL, Miller KD, Jemal A (2015). Cancer statistics, 2015. CA Cancer J Clin.

[2] Bookman MA (2005). Standard treatment in advanced ovarian cancer in 2005: the state of the art. Int J Gynecol Cancer.

[3] du Bois A, Luck HJ, Meier W, Adams HP, Mobus V, Costa S (2003). A randomized clinical trial of cisplatin/paclitaxel versus carboplatin/paclitaxel as first-line treatment of ovarian cancer. J Natl Cancer Inst.

[4] Ozols RF, Bundy BN, Greer BE, Fowler JM, Clarke-Pearson D, Burger RA (2003). Phase III trial of carboplatin and paclitaxel compared with cisplatin and paclitaxel in patients with optimally resected stage III ovarian cancer: a Gynecologic Oncology Group study. J Clin Oncol.

[5] Chu CS, Kim SH, June CH, Coukos G (2008). Immunotherapy opportunities in ovarian cancer. Expert Rev Anticancer Ther.

[6] Leffers N, Gooden MJ, de Jong RA, Hoogeboom BN, ten Hoor KA, Hollema H (2009). Prognostic significance of tumor-infiltrating T-lymphocytes in primary and metastatic lesions of advanced stage ovarian cancer. Cancer Immunol Immunother.

[7] Sato E, Olson SH, Ahn J, Bundy B, Nishikawa H, Qian F (2005). Intraepithelial CD8+ tumor-infiltrating lymphocytes and a high CD8+/regulatory T cell ratio are associated with favorable prognosis in ovarian cancer. Proc Natl Acad Sci U S A.

[8] Sadelain M, Riviere I, Brentjens R (2003). Targeting tumours with genetically enhanced T lymphocytes. Nat Rev Cancer.

[9] Moon EK, Wang LC, Dolfi DV, Wilson CB, Ranganathan R, Sun J (2014). Multifactorial T-cell hypofunction that is reversible can limit the efficacy of chimeric antigen receptor-transduced human T cells in solid tumors. Clin Cancer Res.

[10] Bast RC, Feeney M, Lazarus H, Nadler LM, Colvin RB, Knapp RC (1981). Reactivity of a monoclonal antibody with human ovarian carcinoma. J Clin Invest.

[11] Yin BW, Dnistrian A, Lloyd KO (2002). Ovarian cancer antigen CA125 is encoded by the MUC16 mucin gene. Int J Cancer.

[12] Kabawat SE, Bast RC, Bhan AK, Welch WR, Knapp RC, Colvin RB (1983). Tissue distribution of a coelomic-epithelium-related antigen recognized by the monoclonal antibody OC125. Int J Gynecol Pathol.

[13] Wang Y, Cheon DJ, Lu Z, Cunningham SL, Chen CM, Luo RZ (2008). MUC16 expression during embryogenesis, in adult tissues, and ovarian cancer in the mouse. Differentiation.

[14] Pai VC, Glasgow BJ (2010). MUC16 as a sensitive and specific marker for epithelial downgrowth. Arch Ophthalmol.

[15] Colombo MP, Trinchieri G (2002). Interleukin-12 in anti-tumor immunity and immunotherapy. Cytokine Growth Factor Rev.

[16] Curtsinger JM, Lins DC, Mescher MF (2003). Signal 3 determines tolerance versus full activation of naive CD8 T cells: dissociating proliferation and development of effector function. J Exp Med.

[17] Broderick L, Brooks SP, Takita H, Baer AN, Bernstein JM, Bankert RB (2006). IL-12 reverses anergy to T cell receptor triggering in human lung tumor-associated memory T cells. Clin Immunol.

[18] Kilinc MO, Aulakh KS, Nair RE, Jones SA, Alard P, Kosiewicz MM (2006). Reversing tumor immune suppression with intratumoral IL-12: activation of tumor-associated T effector/memory cells, induction of T suppressor apoptosis, and infiltration of CD8+ T effectors. J Immunol.

[19] Watkins SK, Egilmez NK, Suttles J, Stout RD (2007). IL-12 rapidly alters the functional profile of tumor-associated and tumor-infiltrating macrophages *in vitro* and *in vivo*. J Immunol.

[20] Santos EB, Yeh R, Lee J, Nikhamin Y, Punzalan B, Punzalan B (2009). Sensitive *in vivo* imaging of T cells using a membrane-bound Gaussia princeps luciferase. Nat Med.

[21] Wang X, Chang WC, Wong CW, Colcher D, Sherman M, Ostberg JR (2011). A transgene-encoded cell surface polypeptide for selection, *in vivo* tracking, and ablation of engineered cells. Blood.

[22] Koneru M, Purdon T, Spriggs D, Koneru S, Brentjens R. IL-12 secreting tumor-targeted chimeric antigen receptor T cells eradicate ovarian tumors *in vivo*. Oncoimmunology. doi:10.4161/2162402X.2014.994446. 10.4161/2162402X.2014.994446 PMC4404840 25949921

[23] Dharma Rao T, Park KJ, Smith-Jones P, Iasonos A, Linkov I, Soslow RA (2010). Novel monoclonal antibodies against the proximal (carboxy-terminal) portions of MUC16. Appl Immunohistochem Mol Morphol.

[24] Markman M, Walker JL (2006). Intraperitoneal chemotherapy of ovarian cancer: a review, with a focus on practical aspects of treatment. J Clin Oncol.

[25] Chekmasova AA, Brentjens RJ (2010). Adoptive T cell immunotherapy strategies for the treatment of patients with ovarian cancer. Discov Med.

[26] Esselen KM, Rodriguez N, Growdon W, Krasner C, Horowitz NS, Campos S (2012). Patterns of recurrence in advanced epithelial ovarian, fallopian tube and peritoneal cancers treated with intraperitoneal chemotherapy. Gynecol Oncol.

[27] Tanner EJ, Black DR, Zivanovic O, Kehoe SM, Dao F, Konner JA (2012). Patterns of first recurrence following adjuvant intraperitoneal chemotherapy for stage IIIC ovarian cancer. Gynecol Oncol.

[28] Ferrand C, Robinet E, Contassot E, Certoux JM, Lim A, Herve P (2000). Retrovirus-mediated gene transfer in primary T lymphocytes: influence of the transduction/selection process and of *ex vivo* expansion on the T cell receptor beta chain hypervariable region repertoire. Hum Gene Ther.

[29] Davila ML, Riviere I, Wang X, Bartido S, Park J, Curran K (2014). Efficacy and toxicity management of 19-28z CAR T cell therapy in B cell acute lymphoblastic leukemia. Sci Transl Med.

[30] Alvarez RD, Sill MW, Davidson SA, Muller CY, Bender DP, DeBernardo RL (2014). A phase II trial of intraperitoneal EGEN-001, an IL-12 plasmid formulated with PEG-PEI-cholesterol lipopolymer in the treatment of persistent or recurrent epithelial ovarian, fallopian tube or primary peritoneal cancer: a gynecologic oncology group study. Gynecol Oncol.

[31] Lenzi R, Edwards R, June C, Seiden MV, Garcia ME, Rosenblum M (2007). Phase II study of intraperitoneal recombinant interleukin-12 (rhIL-12) in patients with peritoneal carcinomatosis (residual disease < 1 cm) associated with ovarian cancer or primary peritoneal carcinoma. J Transl Med.

[32] Brentjens RJ, Davila ML, Riviere I, Park J, Wang X, Cowell LG (2013). CD19-targeted T cells rapidly induce molecular remissions in adults with chemotherapy-refractory acute lymphoblastic leukemia. Sci Transl Med.

[33] Diaz-Buxo JA, Chandler JT, Farmer CD, Walker PJ (1980). Intraperitoneal infusion of non-absorbable steroids in the treatment of ascites and sterile peritonitis. J Dial.

[34] Jenkin RP, Bamford R, Patel V, Kelly L, Stern S (2008). The use of intraperitoneal triamcinolone acetonide for the management of recurrent malignant ascites in a patient with non-Hodgkin’s lymphoma. J Pain Symptom Manage.

[35] Mackey JR, Wood L, Nabholtz J, Jensen J, Venner P (2000). A phase II trial of triamcinolone hexacetanide for symptomatic recurrent malignant ascites. J Pain Symptom Manage.

[36] Wolchok JD, Hoos A, O’Day S, Weber JS, Hamid O, Lebbe C (2009). Guidelines for the evaluation of immune therapy activity in solid tumors: immune-related response criteria. Clin Cancer Res.

[37] Chinnasamy D, Yu Z, Kerkar SP, Zhang L, Morgan RA, Restifo NP (2012). Local delivery of interleukin-12 using T cells targeting VEGF receptor-2 eradicates multiple vascularized tumors in mice. Clin Cancer Res.

[38] Chmielewski M, Kopecky C, Hombach AA, Abken H (2011). IL-12 release by engineered T cells expressing chimeric antigen receptors can effectively Muster an antigen-independent macrophage response on tumor cells that have shut down tumor antigen expression. Cancer Res.

[39] Zhang L, Morgan RA, Beane JD, Zheng Z, Dudley ME, Kassim SH, et al*.* Tumor infiltrating lymphocytes genetically engineered with an inducible gene encoding Interleukin-12 for the immunotherapy of metastatic melanoma. Clin Cancer Res. 2015 Feb 18. [epub ahead of print] doi: 10.1158/1078-0432.CCR-14-2085. 10.1158/1078-0432.CCR-14-2085 PMC4433819 25695689

[40] Kershaw MH, Westwood JA, Parker LL, Wang G, Eshhar Z, Mavroukakis SA (2006). A phase I study on adoptive immunotherapy using gene-modified T cells for ovarian cancer. Clin Cancer Res.

[41] Barber A, Zhang T, DeMars LR, Conejo-Garcia J, Roby KF, Sentman CL (2007). Chimeric NKG2D receptor-bearing T cells as immunotherapy for ovarian cancer. Cancer Res.

[42] Carpenito C, Milone MC, Hassan R, Simonet JC, Lakhal M, Suhoski MM (2009). Control of large, established tumor xenografts with genetically retargeted human T cells containing CD28 and CD137 domains. Proc Natl Acad Sci U S A.

[43] Song DG, Ye Q, Carpenito C, Poussin M, Wang LP, Ji C (2011). *In vivo* persistence, tumor localization, and antitumor activity of CAR-engineered T cells is enhanced by costimulatory signaling through CD137 (4-1BB). Cancer Res.

